# Assessing Errors Inherent in OCT-Derived Macular Thickness Maps

**DOI:** 10.1155/2011/692574

**Published:** 2011-08-17

**Authors:** Daniel Odell, Adam M. Dubis, Jackson F. Lever, Kimberly E. Stepien, Joseph Carroll

**Affiliations:** ^1^Department of Ophthalmology, Medical College of Wisconsin, Milwaukee, WI 53226, USA; ^2^Department of Cell Biology, Neurobiology, & Anatomy, Medical College of Wisconsin, Milwaukee, WI 53226, USA; ^3^Department of Ophthalmology, William Beaumont Hospital, Royal Oak, MI 48073, USA; ^4^Department of Biophysics, Medical College of Wisconsin, Milwaukee, WI 53226, USA

## Abstract

SD-OCT has become an essential tool for evaluating macular pathology; however several aspects of data collection and analysis affect the accuracy of retinal thickness measurements. Here we evaluated sampling density, scan centering, and axial length compensation as factors affecting the accuracy of macular thickness maps. Forty-three patients with various retinal pathologies and 113 normal subjects were imaged using Cirrus HD-OCT. Reduced B-scan density was associated with increased interpolation error in ETDRS macular thickness plots. Correcting for individual differences in axial length revealed modest errors in retinal thickness maps, while more pronounced errors were observed when the ETDRS plot was not positioned at the center of the fovea (which can occur as a result of errant fixation). Cumulative error can exceed hundreds of microns, even under “ideal observer” conditions. This preventable error is particularly relevant when attempting to compare macular thickness maps to normative databases or measuring the area or volume of retinal features.

## 1. Introduction

Optical coherence tomography (OCT) provides high-resolution, cross-sectional tomographic images of the human retina and permits direct evaluation of retinal thickness [1]. In recent years the development of spectral-domain OCT (SD-OCT) technology has greatly increased imaging speed and resolution relative to earlier time-domain technology. SD-OCT has become invaluable in the management of a variety of retinal diseases including neovascular age-related macular degeneration (AMD) [2–5] and diabetic macular edema [6, 7]. This utility is due primarily to the ability to extract estimates of retinal thickness across the macula (to aid in clinical diagnosis and treatment decisions). 

Previous studies on the application of SD-OCT to retinal pathology have uncovered multiple sources of error that dramatically decrease the accuracy of these macular thickness measurements [8, 9]. Perhaps the most obvious source of error is imprecise retinal layer segmentation, which can result from poor signal quality of the SD-OCT image or the outright failure in the segmentation algorithm itself in otherwise high-quality images [8, 10, 11]. Additional errors inherent to the system can be elucidated by evaluating the reproducibility of SD-OCT systems [9, 12–17]. These reproducibility studies capture all errors inherent to the basic operation of the SD-OCT system and represent a baseline level of error that could reasonably be expected even under the best circumstances. 

However, there are additional sources of inaccuracy that have received considerably less attention and are independent of segmentation and operator errors. Rather they pertain to instrument sampling and processing protocols. For example, Sadda et al. compared central subfield thickness values from volumes containing 128 B-scans to less densely sampled volumes [18]. As B-scan density is reduced, less retinal area is sampled, leading to less data being included in the retinal thickness calculation. The reduction in data led to differences, or errors, in retinal thickness measurements, the magnitude of which increased as sampling density was decreased [18]. Here, we further examined B-scan density as well as factors that are related to assumptions about the patient being imaged, such as errant fixation and variation in axial length among patients. Taken together, these variables compromise the accuracy of macular thickness maps. While the degree of inaccuracy depends on the patient, the significance of the inaccuracy depends on the application of the retinal thickness data.

## 2. Materials and Methods

### 2.1. Subjects

One hundred thirteen normal subjects (55 male, 58 female) age 18 years and older were recruited for SD-OCT imaging (mean ± standard deviation = 27.3 ± 8.3 years). Normal subjects had normal color vision assessed with the Neitz Test of Color Vision [19] and no history of refractive surgery or any vision-limiting ocular pathology. Forty-three patients (18 male, 25 female) with various retinal pathologies were also recruited (mean ± standard deviation = 40.7 ± 20.1 years). Pathology included macular dystrophy (*n* = 9), blue cone monochromacy (*n* = 3), X-linked high myopia (*n* = 4), basal laminar drusen (*n* = 5), retinitis pigmentosa (*n* = 2), AMD (*n* = 3), plaquenil toxicity (*n* = 3), diabetic macular edema (*n* = 3), macular telangectasia (*n* = 2), central artery occlusion (*n* = 2), and one each of oligocone trichromacy, posterior epithelial detachment, oculocutaneous albinism, punctate inner choroidopathy, achromatopsia, cystoid macular edema, and acute zonal occult outer retinopathy. Informed consent was obtained from all subjects after explanation of the nature and possible consequences of the study. All research on human subjects followed the tenets of the Declaration of Helsinki and was approved by the Institutional Review Board at Children's Hospital of Wisconsin.

### 2.2. SD-OCT Retinal Imaging

Volumetric SD-OCT images of the macula were obtained using the Cirrus HD-OCT (Carl Zeiss Meditec, Dublin, Calif, USA). Volumes were nominally 6 mm × 6 mm and consisted of 128 B-scans (512 A-scans/B-scan). The internal fixation target of the system was used, which consists of a large green asterisk on a red background, and focus of the LSO fundus image was optimized using built-in focus correction. In addition, the polarization setting was optimized using the built-in function for each eye. Retinal thickness was calculated using the built-in Macular Analysis software on the Cirrus (software version 5.0), which is automatically determined by taking the difference between the ILM and RPE boundaries [20]. The positions of the foveal center and retinal thickness data from each volume scan were exported for offline analysis using the Zeiss Cirrus Research Browser (version 5.0). All volumes were manually examined for accuracy of the ILM and RPE segmentation and relative accuracy of the Autofovea function.

### 2.3. Manipulation of Macular Thickness Maps

**Figure 1:**
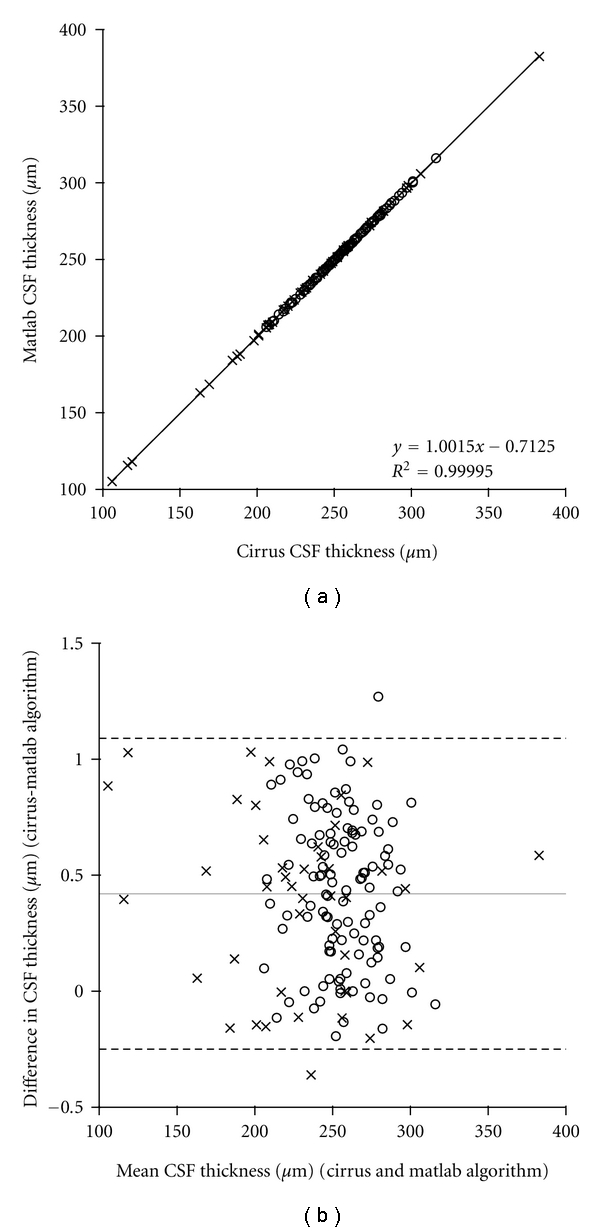
A custom MatLab program was designed to generate ETDRS thickness plots from the raw thickness data exported using the Cirrus Research Browser. (a) Central subfield thickness values taken directly from the Cirrus correlate highly with those obtained from our Matlab-based algorithm. (b) Bland-Altman plot further reveals excellent agreement between the two measurements. Gray line represents the mean difference between the measurements (0.42 *μ*m), and the dashed lines represent 95% confidence limits (1.09 *μ*m and −0.25 *μ*m). These data indicate virtually no loss in accuracy when using the MatLab derived thickness maps for subsequent analysis. *Open circles*: normals; *crosses*: pathology subjects; CSF = central subfield.

In order to evaluate the acquisition and analysis parameters of interest, we needed to be able to manipulate these macular thickness maps off line. Custom Matlab (Mathworks, Natick, Mass, USA) software was used to generate early treatment diabetic retinopathy study (ETDRS) thickness maps from the  .dat files exported from the Zeiss Cirrus Research Browser. As shown in Figure 1, there is good agreement between ETDRS segment thicknesses derived from the on-board Cirrus software and our offline Matlab program, thus demonstrating the fidelity of the data export and validating our use of these Matlab-derived ETDRS maps for subsequent analysis.

To assess the interpolation error in volumetric retinal thickness maps due to decreased B-scan sampling, we created undersampled versions of the retinal thickness volumes exported from the Cirrus system. These maps used thickness values from 8 (every 16th B-scan), 16 (every 8th B-scan), 32 (every 4th B-scan), or 64 (every other B-scan) of the 128 B-scans initially collected. Complete thickness maps were then created by interpolating between these evenly spaced B-scans (using a Matlab spline interpolation function). This enabled point-by-point comparison between the native macular thickness map and the undersampled ones, as well as comparison between the corresponding ETDRS plots. In all ETDRS comparisons, mean differences were computed using absolute differences.

Most SD-OCT systems assume foveal fixation; however there is frequently significant discrepancy between the location of the fovea and the preferred retinal locus of fixation. Even among individuals with no retinal pathology, there is modest variation in fixation and there is evidence that suggests that the foveal center is not always used for fixation [21–24]. We used the Autofovea function of the Cirrus HD-OCT to identify the location of the foveal pit and generated an ETDRS plot centered at this location and a second plot centered at the middle of the volume (the default setting on most other SD-OCT systems). Manual inspection of each volume confirmed that the fovea was identified by the Autofovea function (though in more severe macular pathology we have seen the algorithm fail). Comparing these two ETDRS plots provides an estimate of the potential error due to improper anchoring of the plot to the scan center. Moreover, as we had access to the (*x*, *y*) coordinate of the fovea within each nominal 6 mm × 6 mm volume, we examined error as a function of the displacement of each subject's fixation from the center of his or her foveal pit.

The scan length reported by SD-OCT systems (when reported in mm) is relative, not absolute. This is because the scanning mirrors are calibrated to a model eye, which assumes a fixed axial length (typically around 24 mm). However there exist significant individual differences in retinal magnification (primarily caused by differences in axial length); thus the actual scan length will vary from person to person. In fact, using normative axial length data [25] to correct for ocular magnification [26], we estimate that approximately one-third of individuals would have a scan length that deviates by more than 0.3 mm from the expected length (with a maximum deviation of nearly 1 mm). We obtained axial length measurements using the Zeiss IOL Master (Carl Zeiss Meditec, Dublin, Calif, USA) and subsequently calibrated the lateral scale of each subject's SD-OCT scans in order to generate revised ETDRS plots. These plots were then compared to those derived assuming a 24.46 mm axial length (that of the Cirrus model eye). 

## 3. Results

### 3.1. Effect of B-Scan Sampling Density on Accuracy of Retinal Thickness Measurements

**Figure 2:**
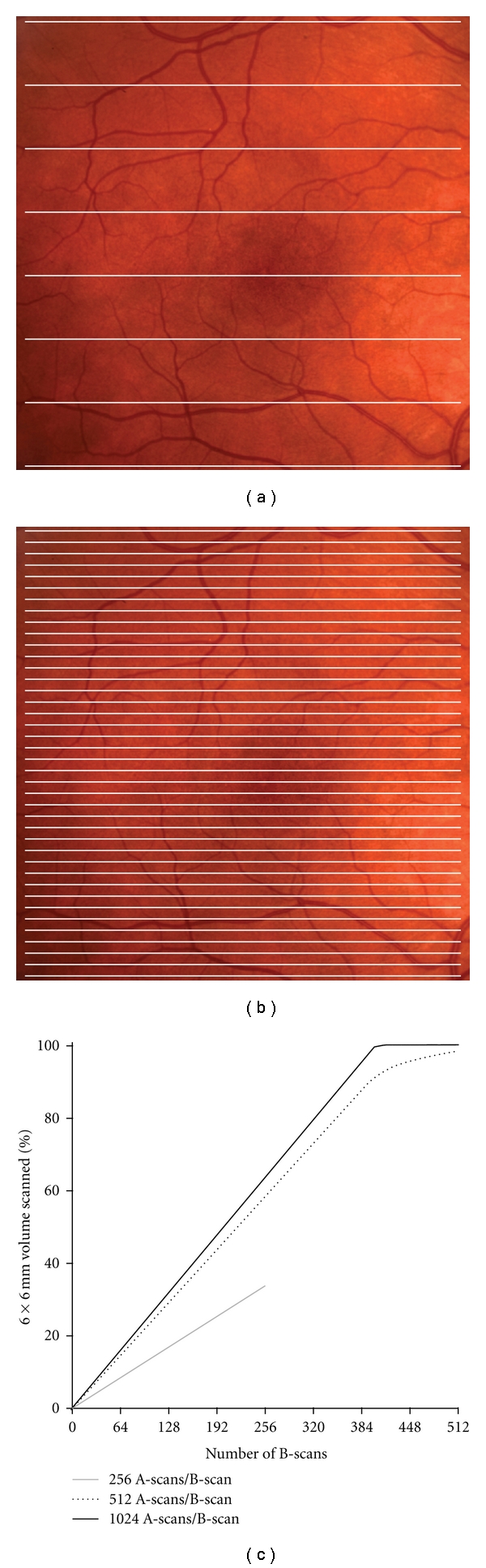
Reduction in B-scan sampling results in less retinal area being scanned. (a) Eight simulated B-scans spaced 750 *μ*m apart over a 6 mm × 6 mm volume. At this sampling, assuming a 15 *μ*m spot size and 512 A scans/B-scan, only 1.8% of the volume is scanned. (b) Sixty-four simulated B-scans spaced 94 *μ*m apart over a 6 mm × 6 mm volume. At this sampling, only 15% of the volume is scanned. (c) Percentage of OCT volume scanned as a function of B-scan density. Complete sampling of the retinal volume at 512 A scans/B-scan would require nearly 600 B-scans.

Despite the macular volume scan nominally subtending a 6 mm × 6 mm area, the entire retinal area within that volume is not actually scanned. As shown in Figure 2, even a scan using 512 A-scans/B-scan and 128 B-scans only samples 29% of the retinal area within the volume. Using 37 high-resolution B-scans results in less than 10% of the retinal area within the volume actually being scanned. As only retina that gets scanned can actually contribute to plots of retinal thickness measurements, this undersampling can significantly affect the integrity of the resultant macular thickness maps.

**Figure 3:**
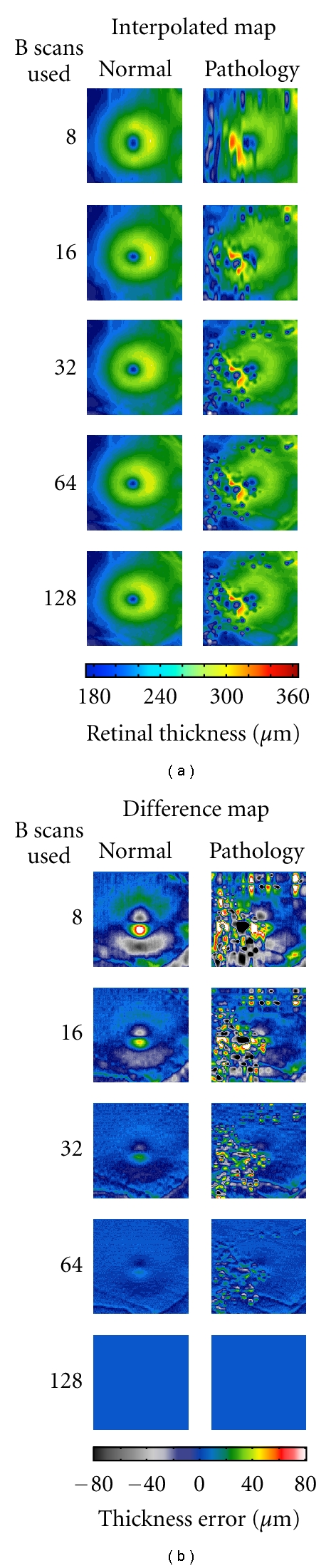
Two examples of how interpolation between B-scans results in inaccurate macular thickness maps. (a) Undersampled thickness maps from a single normal subject and one with dominant drusen retain relatively similar qualitative appearance to that of their respective 128 B-scan maps. (b) The thickness differences between the standard of 128 scans and each sequential level of B-scan density show the areas of the macula most effected by undersampling in a normal subject and a subject with dominant drusen. Even with 32 B-scans significant error is generated through the central fovea (where the contour is changing most rapidly) in a normal subject. This effect is greatly enhanced in the subject with drusen wherein error is generated not only in the central fovea but also broadly across the entire scanning area.

At first glance, assessment of the effect of B-scan density on macular thickness maps suggests that despite reducing the number of B-scans, the general contour of the map remains qualitatively similar (Figure 3(a)). However in reality, interpolation between B-scans causes overrepresentation and underrepresentation of different features within a given retinal volume (Figure 3(b)). As shown in the normal example, since sampling is being reduced in the vertical direction, the superior and inferior aspects of the fovea show equal magnitude of underrepresentation and overrepresentation of retinal thickness, respectively. This effect is greatly enhanced in a subject with dominant drusen, wherein error is generated not only in the central fovea but also broadly across the retinal volume.

We found that in the normal individuals for the central 1 mm subfield, the mean (± standard deviation) absolute error was 0.19 ± 0.15 *μ*m with 64 B-scans, 1.17 ± 0.69 *μ*m with 32 B-scans, 7.15 ± 2.35 *μ*m with 16 B-scans, and 22.98 ± 7.54 *μ*m with 8 B-scans. When expressed as a percentage of subfield thickness we find that the mean percentage error was 0.07 ± 0.06% with 64 B-scans, 0.47 ± 0.29% with 32 B-scans, 2.85 ± 1.08% with 16 B-scans, and 9.19 ± 3.52% with 8 B-scans. We found similar differences in the individuals with retinal pathology. For the central 1 mm subfield, the mean (± standard deviation) absolute error was 0.31 ± 0.36 *μ*m with 64 B-scans, 1.36 ± 1.17 *μ*m with 32 B-scans, 6.35 ± 3.64 *μ*m with 16 B-scans, and 19.56 ± 11.08 *μ*m with 8 B-scans. When expressed as a percentage of subfield thickness we find that the mean percentage error was 0.13 ± 0.15% with 64 B-scans, 0.63 ± 0.56% with 32 B-scans, 3.03 ± 2.14% with 16 B-scans, and 9.56 ± 6.92% with 8 B-scans. Previous data reveal that the coefficient of repeatability for central subfield measurements on the Cirrus is about 4.96 *μ*m, indicating that 32 B-scans is sufficient sampling to generate accurate ETDRS thickness plots. 

**Figure 4:**
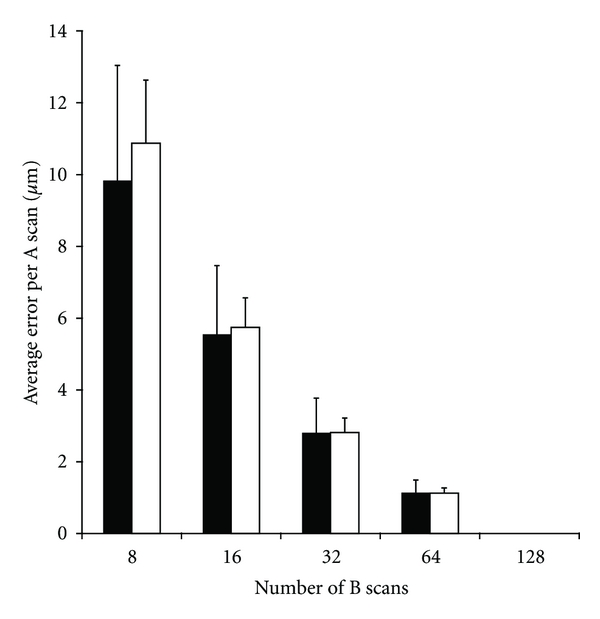
The impact of undersampling for retinal thickness measurements. As fewer B-scans are used, the average error per pixel (A scan) increases. This value was obtained by first subtracting each undersampled macular thickness map from the native 128 B-scan thickness map and then dividing the difference by the number of pixels (A scans).

However, as shown in the difference plots in Figure 3, at neighboring retinal locations where the retinal contour is changing, retinal thickness measurements are in error in opposing directions. Thus, reporting retinal thickness for a subregion that averages spatially (i.e., ETDRS plots) will not reveal the true extent of the error imparted by undersampling. In order to quantify the effect of B-scan density on the accuracy of retinal thickness at any given point within the 6 mm × 6 mm volume, we examined the error per pixel (“A scan”) within the volume. In this case, the retinal thickness measurements utilizing all 128 B-scans were considered to be absolutely accurate for comparison to the undersampled volumes. At 32 B-scans, our analysis revealed that these interpolation errors could be as high as 5.5 *μ*m per pixel and 7.5 *μ*m per pixel in the normal and pathology groups, respectively. There are 65,536 pixels in each of our thickness maps, native or undersampled. In both groups, on average, the error per pixel increases as the number of B-scans used to construct the retinal thickness map decreases (Figure 4).

### 3.2. Position of SD-OCT Volume with Respect to the Fovea and ETDRS Plot Accuracy

**Figure 5:**
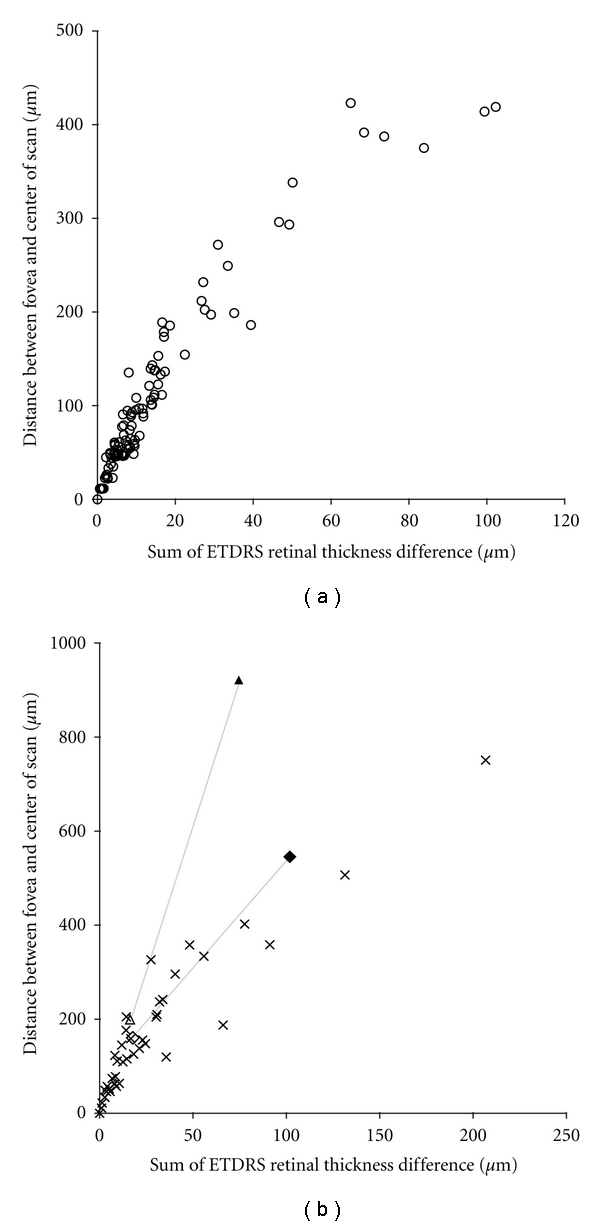
Measuring the effect of scan position on accuracy of ETDRS thickness plots. For each subject, we estimated this error by comparing the raw ETDRS thickness plot to one that has been repositioned to be centered on their fovea. In the normal individuals (a), the summed error across the nine ETDRS segments could be as much as 100 *μ*m, while in individuals with pathology (b) the error could exceed 200 *μ*m. For some pathology cases, nonfoveal fixation can be compensated for by moving the location of the OCT scan. For 2 individuals, we acquired scans at their eccentric fixation location (filled triangle and diamond) and a second scan after the operator manually moved the scan to be centered on the fovea (open triangle and diamond, connected by thin gray lines). Even when using the repositioned scan, residual error remains, though it is on the order of that observed for the other patients.

We compared ETDRS thickness plots derived by placing the center of the ETDRS grid on the foveal center to those plots centered on the subject's actual fixation point. We found these plots to differ by over 100 *μ*m in some normal individuals (sum of the error in all nine ETDRS segments), with the mean error being 14.4 ± 19.3 *μ*m (Figure 5(a)). In the 43 pathology cases, the mean error was 30.4 ± 40.9 *μ*m, with some individuals exceeding 200 *μ*m of total error in their ETDRS plots (Figure 5(b)). Of course if eccentric fixation is identified by the OCT operator, the scan location can be repositioned prior to image acquisition to help reduce this error. For two pathology cases, we acquired one scan at their normal eccentric fixation location and a second after moving the scan to be visually centered on the fovea. At their normal fixation position, these subjects had ETDRS plots that deviated by 74.5 *μ*m and 101.9 *μ*m from an ETDRS plot precisely positioned at the foveal center (using our offline MatLab program). Even after the operator acquired a second scan intentionally centered on the fovea to the best of their ability, ETDRS errors persisted of 16.3 *μ*m and 17.8 *μ*m. Regardless, for both normal subjects and subjects with retinal pathology, the greater the distance between the fovea and the center of the SD-OCT volume, the less accurate the ETDRS thickness map. In just the central subfield thickness, not correcting for scan position results in a mean error of 3.18 ± 6.09 *μ*m in the normal subjects (with a maximum error of 32 *μ*m) and 10.50 ± 19.43 *μ*m in the patients with retinal pathology (with a maximum error of 104 *μ*m). On average, the central subfield error accounts for 14% and 22% of the total ETDRS error in the normal and pathology patients, respectively.

### 3.3. The Effect of Ocular Magnification on ETDRS Plot Accuracy

**Figure 6:**
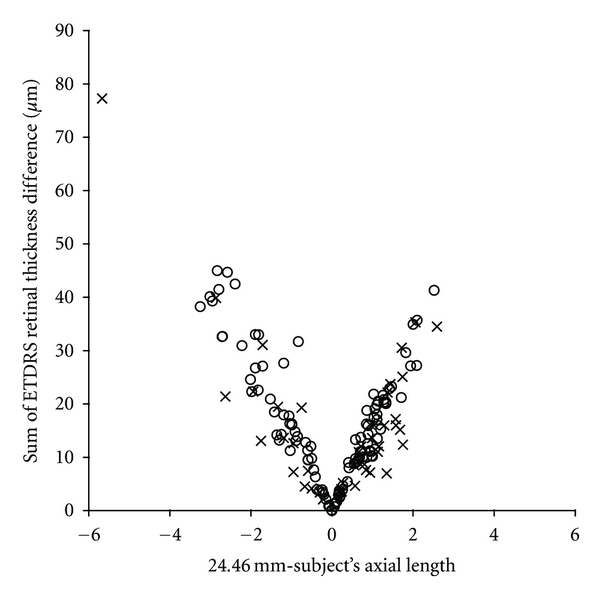
The effect of axial length on the accuracy of ETDRS thickness plots. For each subject, we compared the raw ETDRS thickness plot assuming a 6 mm scan size to an ETDRS thickness plot using a scan length corrected for their axial length. As the deviation in axial length increases, the error in ETDRS thickness plots (sum of all nine ETDRS segments) becomes greater, both for the normal subjects (open circles) and the pathology subjects (crosses).

Axial length varied in our normal subjects from 21.56 to 28.36 mm and in pathology patients from 21.87 to 30.13 mm. Using each subject axial length to correct the lateral scale of the nominal 6 mm SD-OCT scan, we determined that actual scan sizes range from about 5.29 to 6.96 mm for our normal population and 5.36 to 7.4 mm for our pathological population. We used these corrected scan dimensions to derive corrected ETDRS plots, where the rings were actually 1 mm, 3 mm, and 6 mm in diameter. In comparing these plots to the uncorrected ones, we found that the summed error for the nine ETDRS segments was as much as 44.9 *μ*m, with 37 out of 113 (32%) subjects having more than 20 *μ*m of total error. For subjects with retinal pathology the summed error for the nine ETDRS segments was as much as 77.3 *μ*m, and 13 out of 43 (30%) showed more than 20 *μ*m of total error (Figure 6). In just the central subfield alone, the error was as much as 7.86 *μ*m (with an average of 2.56 ± 1.85 *μ*m) for the normals and as much as 12.33 *μ*m (with an average of 2.84 ± 2.46 *μ*m) in the individuals with retinal pathology. In both groups, the error increased with increasing difference in axial length from that of the model eye (24.46 mm).

### 3.4. The Combined Error due to Ocular Magnification and Scan Positioning

As illustrated above, not correcting for axial length and not positioning the scan at the center of the fovea introduces significant error in the corresponding ETDRS thickness plots. Taken together, these artifacts tend to have a cumulative negative effect on the accuracy of the ETDRS plots. For example, in considering just the central subfield thickness, not correcting for axial length or scan position results in a mean error of 4.53 ± 5.77 *μ*m in the normal subjects (with a maximum combined error of 33 *μ*m) and 11.29 ± 19.18 *μ*m in the patients with retinal pathology (with a maximum combined error of 105 *μ*m).

## 4. Discussion

This study examined the effects of preventable operational and analytic aspects of the SD-OCT on the overall accuracy of ETDRS retinal thickness plots. Scan density, position of the scan with respect to the foveal center, and magnitude of subject axial length differential all contribute to significant error in computing retinal thickness from SD-OCT volumes. An important point to consider is the cumulative nature of the errors reported here; these parameters should all be accounted for when developing normative databases or analyzing specific retinal features within individual patient data. While the errors were estimated using a single SD-OCT device (Cirrus HD-OCT), they are generic to SD-OCT imaging in general. The issue of scan positioning is typically something that can be addressed by the operator by repositioning the ETDRS grid (either manually or using an automatic function like Autofovea). Currently, correcting the lateral scale of OCT data/images requires offline correction by the user.

In comparing our results to previously published data, we find similarities and differences. In an examination of B-scan density, Sadda et al. concluded that 32 B-scans result in only a minimal change in retinal thickness [18]. Our data also show that when examining maps of retinal thickness that are based on spatially integrating individual thickness values (i.e., ETDRS), reduced B-scan sampling has minimal impact. However, if interested in deriving absolute measures of retinal thickness at any given point, reduction to 32 B-scans (a value suggested to provide accurate retinal thickness maps), results in an average error of around 3 *μ*m per pixel. While this average error is within the system resolution on commercial SD-OCT systems, it is worth keeping in mind that the error at any one pixel can be much larger, since not all pixels will contribute equally to the total error (which is implicit in computing an average error). We feel this more accurately reflects the “real” cost of undersampling, and this would significantly limit the ability to make precise measurements of retinal features (e.g., drusen). This highlights the importance of considering how the SD-OCT data is going to be used when deciding how densely to sample the retina.

It is well documented that differences in axial length result in different ocular magnification of retinal images and thus can affect the accuracy of measurements of retinal features [27]. With respect to OCT, axial length has been shown to influence measurements of retinal nerve fiber layer (RNFL) thickness [28–31]. This of course is based on the fact that RNFL measures are presumed to be taken at a fixed distance from the optic nerve; thus individual differences in ocular magnificent would result in the RNFL being measured at the wrong location. Here we demonstrate that individual differences in ocular magnification also affect the accuracy of macular thickness maps. If the distribution of axial lengths in a normative database does not match that of the subject population being studied, misinterpretation can occur. Perhaps more important than retinal thickness maps is the fact that not correcting the nominal scan length for differences in axial length will obviate making reliable measurements in the lateral dimension within a given OCT dataset. This could include measuring the area of geographic atrophy, the size of a macular hole, or the size of a druse. Despite this, some SD-OCT systems still output lateral scale bars on their images that are given in *μ*m or provide calipers with which to make lateral measurements in *μ*m, despite no correction for axial length having been made. One should avoid using such scale bars to report absolute length measurements, as they are simply not accurate without first taking into account ocular magnification.

There have also been previous examinations of the effect of fixation on the accuracy of OCT thickness measurements. In glaucoma, it has been shown that if the circular scan is not centered on the ONH, the RNFL thickness measurements are inaccurate [32]. Campbell et al. [33] examined how intentionally shifting the center of macular volume OCT scans (Stratus time-domain) affected central subfield thickness measurements for 10 normal subjects. They found that scan decentration of 0.50 mm resulted in foveal thickness measurements that were in error by about 45%. For our normal subjects, the average decentration of the SD-OCT volume with respect to the foveal center was 0.09 mm and the average error of foveal thickness measurements was about 35%. While this is roughly consistent with the finding of Campbell et al. [33], some discrepancy would be expected given our use of SD-OCT (instead of time domain) and our ability to precisely determine the exact misalignment between the two scans being compared (whereas the previous study would have be confounded by errors due to normal fixational instability). Currently, the Cirrus HD-OCT will automatically position the ETDRS grid over the center of the fovea (after the scan is taken). While this results in a more accurate ETDRS map, it may not be valid to compare these maps to a database in which the ETDRS maps were not centered on the fovea, though in the case of the Cirrus database, good centration of the volume on the fovea was an inclusion criterion. It is generally important to ensure that the scan parameters used to develop the normative database match that of the on-board scan protocol. Moreover, the subject composition (race and gender) may also need to be considered when comparing a specific patient to a particular normative database [12].

There are several limitations to the present study. First, in our examination of B-scan sampling, we used 128 B-scans as the “truth”. This was simply due to a limitation of the specific SD-OCT device being used. However, as we showed in Figure 2, 128 B-scans (at 512 A scans/B-scan) only sample 29% of the nominal 6 m × 6 mm volume. Thus these volumes are likely already in error compared to an isotropic volume of 512 B-scans. With the expected availability of even faster OCT systems, it will be important to quantify the level of inaccuracy systematically across more densely sampled volumes. In addition, we likely underestimate the real effect of undersampling, as we used simulated thickness maps. If one were to really only acquire 32 B-scans, this could affect the accuracy of segmentation as many OCT devices use 3D approaches to make correct assignment of layers. A second limitation is that we corrected for ocular magnification using a linear scaling based on axial length. There are other methods to correct for ocular magnification [26], and the exact method used for the correction would influence the measured differences in retinal image magnification. Finally, we did not subanalyze different pathologies. It seems likely that different retinal pathology would suffer more (or less) than others. Intuitively, one can conclude that the more uniform the retinal thickness contoured (as might occur in retinitis pigmentosa, where the retina is uniformly thin), the less impact the B-scan sampling, axial length, and scan position would have. Likewise, retinal pathology that results in significant peaks and troughs in retinal thickness (macular holes, AMD, diabetic macular edema) might be more significantly influenced by these parameters. A more detailed, disease-specific analysis is required to clarify this issue.

It is important to keep in mind that the relevance of these errors of course ultimately depends on the clinical application. For monitoring patients over time, relative differences in retinal thickness would be generally unaffected by axial length, though comparing populations of patients (such as in a clinical trial) where there may be differences in axial length between the groups could result in significant error. If one uses the same sampling density, then the accuracy of these longitudinal measurements of retinal thickness will be on the order of that reported for previous repeatability and reproducibility studies. However, in instances where one is interested in correlating a measure of retinal thickness over a specific retinal area (e.g., central subfield thickness) with some other measure of vision (such as treatment response) these errors could reveal correlations that do not exist or hide ones that do exist. Moreover, where one is interested in making absolute measurements in the lateral dimension, such as foveal pit morphology [12, 34] or drusen volume [35], it is critical that these sources of error be removed.
